# Quality, proximate composition, and sensory characteristics of Dorper, domestic commercial crossbred, and Australian sheep meat: a comparative study

**DOI:** 10.1093/tas/txab024

**Published:** 2021-02-09

**Authors:** K M Villatoro, F Yang, T Duarte, C R Phillips, D R Woerner, M D Chao, X Yang

**Affiliations:** 1 Department of Animal Science, University of California, Davis, USA; 2 College of Agriculture, California State University, Chico, USA; 3 Department of Animal and Food Sciences, Texas Tech University, Lubbock, USA; 4 Department of Animal Sciences and Industry, Kansas State University, Manhattan, USA

**Keywords:** consumer panel, Dorper, meat quality, sheep, trained panel

## Abstract

The objective of this study was to compare the proximate, quality, and sensory attributes of Dorper sheep meat (Dorper), domestic commercial crossbred (DCC) and Australian commercial crossbred (ACC). A total of 60 untrimmed loins from the three sheep sources were purchased (20 sheep loins/source) and processed. The objective color, objective tenderness [Warner–Bratzler Shear Force (WBSF)], and proximate composition of the sheep meat were evaluated. A consumer panel and a trained sensory panel were also conducted to evaluate the sensory attributes. Dorper had greater (*P* = 0.04) carbohydrate content compared to DCC, but was not (*P* = 0.86) different from ACC. In addition, Dorper had the greatest WBSF value, followed by DCC, with ACC having the least WBSF out of the three (*P* < 0.0001). For the consumer panel, Dorper was rated to be less tender than ACC (*P* = 0.01), but was not different from DCC (*P* = 0.76). Dorper was also rated with lower flavor acceptability compared to DCC (*P* = 0.02), but was not different from ACC (*P* = 0.86). In addition, Dorper had the lowest overall acceptance rating by the consumers (*P* = 0.01). Trained sensory panel results followed the same trend as the consumer panel results which rated Dorper to be less tender than ACC (*P* = 0.002), but was not different from DCC (*P* = 0.10). Dorper was also rated with greater off-flavor intensity compared to DCC (*P* = 0.009), but was not different from ACC (*P* = 0.53). Finally, no differences were found for all other attributes evaluated among the sheep sources. The results indicated that consumers did not prefer Dorper over ACC and DCC. However, additional research with a more controlled environment is needed to shed light on the true palatability traits of Dorper.

## INTRODUCTION

Lamb is expensive (US$20.77 /kg) in comparison to other red meats such as pork (US$9.19/kg) and beef (US$14.65/kg.; USDA-ERS, 2016). The consumption of lamb varies in magnitude depending on socio-economic factors, religious beliefs, cultural practices, sensory attributes, and marketing factors (Font-i-Furnols and Guerrero, 2014). The U.S. sheep industry has faced a decline in inventory since the mid-1940s, from 56 million in 1942 to 4 million heads in 2004 due to decline in demand as well as increased competition from foreign lamb meat (Jones, 2004). About half of the retail lamb products in the U.S. are imported from other countries, and 70% of these imports comes from Australia as Australian lamb meat is known to be economical and highly acceptable in meat quality traits (Russell et al., 2005; USDA-ERS, 2019; O’Reilly et al., 2020).

The Dorper lamb breed has been raised in different countries, such as the United States, Brazil, Ethiopia, South Africa, and China (Cheng, 1984; Canton et al., 2009; Deng et al., 2012; Yeaman et al., 2013; Ma et al., 2014). Previous studies have shown that the Dorper breed is adaptable to the harsh environment(s), fast-growing, and produces heavy carcasses that result in more attractive cuts for consumers and retailers (Basson et al., 1969; Manyuchi et al., 1991; Shackelford et al., 2012; Souza et al., 2016). The Dorper sheep breed was imported into the United States in the early 1990s (Snowder and Duckett, 2003) and is gaining popularity in the United States potentially due to its meat quality attributes (Shackelford et al., 2012). However, little research has been done to compare the quality traits of lamb meat from Dorpers and other common sources of lamb meat sold in the United States (Clarke et al., 1996; Duckett and Kuber, 2001; Shackelford et al., 2012).

In response to the decline in demand and increased foreign competition, U.S. sheep industries must reply with consistent production of a uniform, safe, nutritious product of exceptional quality that meets consumer expectations. The future of the U.S. sheep industry depends on the demand and profitability of lamb, and the Dorper sheep breed may be able to improve the sheep industry with its potential sensory advantages over the other breeds. Therefore, the primary objective of this research is to evaluate the proximate composition, quality, and sensory traits of Dorper sheep meat (Dorper) compared to sheep meat from domestic commercial crossbred (DCC) and Australian commercial crossbred (ACC).

## MATERIALS AND METHODS

### Sample Collection and Preparation

Sixty whole loins (NAMP #231) from Dorper (*n* = 20), DCC (*n* = 20), and ACC (*n* = 20) were purchased from a processing plant in Texas, a processing plant in California and a warehouse in California, respectively. The average loin weighed approximately 3.13 kg (Dorper), 3.08 kg (DCC), and 1.95 kg (ACC). The loins were vacuum packaged and shipped in refrigerated conditions (2 ± 2 ˚C) to California State University-Chico Meats Laboratory. Loins from all sheep sources were aged in a cooler (2 ± 2 ˚C) according to their production dates to achieve an aging time between 29 and 32 d. All aged loins were frozen (−20 ˚C) until sample preparation.

Loins from all sheep sources were removed from the freezer and thawed in a cooler (2 ± 2 ˚C) for 48 h. After thawing, each loin was split and cut on a bandsaw into 2.54 cm chops. The loin was further deboned and trimmed to 0.30 cm subcutaneous fat. The first chop from the anterior end of the left side was further trimmed to remove all subcutaneous fat and designated for proximate analysis. The next three chops from the left side were designated for quality traits analysis (pH, objective color, Warner–Bratzer Shear Force [WBSF], and cooking loss). The first four chops from the anterior end of the right side were designated for consumer panel analysis, and the next three chops were designated for trained panel analysis. All chops were vacuum-packaged and immediately frozen (–20 ˚C) prior to the analysis.

### Quality Traits Analysis

The pH of each sheep meat sample was measured in duplicate using a hand-held pH meter with a penetrating glass electrode (WD-35634-30, Oakton Instruments, Vernon Hills, IL, USA) at the geometric center of the chop. The pH meter was calibrated using pH 10.0, 7.0, and 4.0 buffers prior to the measurement. The pH electrode was rinsed with distilled water and wiped dry between samples using Kimwipes (Kimberly-Clark, Irving, TX, USA).

For objective color evaluation, chops were allowed to bloom for 30 min, and the color of the chops (lean portion only) was measured using CIE L*(lightness), a*(redness), and b*(yellowness) system with a colorimeter (CR-400, Minolta, Osaka, Japan) set at a D65 light source and 2° observer with an 8-mm diameter measurement area. The colorimeter was calibrated using a white ceramic tile provided by the manufacturer. The mean of six random readings through the polyvinyl chloride film from the cut surface of the three chops designated for quality traits analysis was recorded for each loin.

For objective tenderness measurement (WBSF), two chops from each loin were cooked on a George Foreman clamshell grill (Spectrum Brands, Middleton, WI, USA) to an internal temperature of 71 °C. The temperature of the chops was monitored with a thermocouple thermometer probe (35100 AquaTuff, Cooper Atkins, Cincinnati, OH, USA), inserted horizontally to the center of the chop. The chops were weighed before and after cooking, and the cooking loss was calculated as the percentage weight loss of each chop before and after cooking. The cooked chops were cooled at 2 ± 2 °C for 24 h prior to the shear force analysis. Three 1.27 cm diameter cores were removed from the *longissimus dorsi* parallel to the muscle fiber using a drill press from each chop. A total of six cores from two chops were obtained for WBSF from each loin. Cores were sheared using a Texture Analyzer (TMS-PRO, Food Technology Corp., Sterling, VA, USA) equipped with a Warner–Bratzler blade with a crosshead speed set at 250 mm/min. The mean of the peak shear force (kg) of six cores was calculated for each loin (USDA-ARS, 2015; American Meat Science Association, 2019).

### Proximate Composition Analysis

Chops designated for proximate composition analysis were shipped to Midwest Laboratories, Inc. (Omaha, NE). The AOAC protocols (1997) were followed by the laboratory to measure the moisture (AOAC 950.46), protein (AOAC 990.03), fat (AOAC 991.36), and ash (AOAC 900.02a). Carbohydrates were calculated as “carbohydrates by difference” as described by Monro and Burlingame (1996) and USDA (2015). Calories were calculated using the Atwater factors by multiplying the grams of protein and carbohydrate in a serving by 4 and the grams of fat by 9 (Merrill and Watt, 1973). The nutritional analysis was performed based on a serving size of 100 grams.

### Consumer Tasting Panel Evaluation

For consumer sensory evaluation (IRB 1112548-1), chops were thawed at 4 °C for 24 h and cooked using identical procedures to those described for WBSF. Immediately after cooking, each chop was cut into half. Samples were placed in glass bowls and covered with aluminum foil marked with random four-digit codes. Samples were kept in an insulated food carrier (Carlisle model PC300N03, Oklahoma, OK, USA) at 60 °C for no longer than 30 min prior to a session. Each tasting sample was then also assigned a random three-digit code and served to consumers in random order simultaneously, ensuring each consumer received a sample from each group.

A total of 120 consumers participated in the consumer evaluation over five sessions. Consumer inclusion criteria required participants between the ages of 18 and 65 yr old with diets that include sheep meat. At the time of each session, consumers filled an individual survey that included information about gender identity, race of origin, age, education level, household size, household income, meat consumption over time, sheep meat consumption over time, and the most important factor influencing the decision to purchase. Each consumer evaluated four samples per session. As a result, each sample was evaluated by eight consumers. Consumers evaluated tenderness, flavor, juiciness, and overall acceptance using a 9-point hedonic scale (1 = dislike extremely, 2 = dislike very much, 3 = dislike moderately, 4 = dislike slightly, 5 = neither like nor dislike, 6 = like slightly, 7 = like moderately, 8 = like very much, and 9 = like extremely). Each consumer received unsalted saltine-type crackers and distilled water for palate cleansing between samples.

### Trained Sensory Evaluation

Chops designated for trained sensory evaluation were shipped frozen overnight to the Colorado State University Meat Laboratory (Fort Collins, CO, USA) where they were stored frozen (–20 °C) until further analysis. For the trained evaluation, chops were thawed for 12 h under refrigeration (0–2 °C). The raw chops were spaced 10 cm apart directly onto the flat side of a non-stick coated grilling plate (Rational, TriLax Model 60.71.617) and cooked to an internal temperature of 62.8 °C using a combination oven (Model SCC WE 61 E; Rational, Landsberg am Lech, Germany) set at 204 °C on default fan speed and 0% humidity. The internal temperature was monitored by placing a K Thermocouple Thermometer (AccuTuff 340, model 34040, Cooper-Atkins Corporation, Middlefield, CT, USA) into the geometric center of each sample. Immediately after cooking, samples were held in a warming oven at 60°C for no more than 30 min prior to serving to panelists. Each sample without external fat was cut into uniform cuboidal sections (1.3 cm × 1.3 cm × 2.54 cm) and served to a minimum of six qualified panelists. Panelists were trained and qualified to objectively quantify: lamb flavor intensity, off-flavor intensity, and tenderness using a 100 mm unstructured line scale anchored at both ends (0 = absence or low intensity of a specified attribute, 100 = extreme intensity of a specified attribute). Additionally, panelists identified and recorded a description of the off-flavors. Each panelist received two to three cuboidal sections or pieces from 10 individual chops over the course of 1 h. Each panelist was seated in a private booth equipped with red incandescent lighting to mask color differences of the samples. Two panels were conducted per day, one in the morning and one in the afternoon. Panelists were supplied with unsalted saltine-type crackers, apple juice, and distilled water for palate cleansing between samples.

### Statistical Analyses

The experimental design of the study was a completely randomized design with fixed effects of the three sheep sources. The experimental units were the individual loins (*n* = 60). One-way analysis of variance (ANOVA) was performed to compare the means of the quality traits and nutritional composition among the three sheep sources using Proc Mixed in SAS (SAS version 9.4, SAS Institute Inc., Cary, NC, USA). Data for trained sensory evaluation were analyzed as a randomized complete block design with each session as the block using Proc Glimmix in SAS. The results were reported as the least-squares means. Nonparametric data from the consumer evaluation were analyzed by the Kruskal–Wallis test using the Proc NPAR1WAY procedures in SAS. The Dwass–Steel–Critchlow–Fligner (DSCF) post hoc test was used after the Kruskal-Wallis test for multiple paired comparisons. An *α* value of 0.05 was used to determine the statistical significance of all the analyses.

## RESULTS AND DISCUSSION

### Quality Traits Analysis

Meat quality results are provided in Table 1. The pH values were different among the three sheep sources (*P =* 0.0007; Table 1). The pH value of Dorper was greater (*P =* 0.0018) compared to DCC, but was not different from ACC (*P* = 0.64). The ultimate pH of red meat can be affected by factors such as breed, age, diet, and stress level (England, 2018). Hopkins et al. (2007) found that the age differences affected the pH of the *semitendinosus* muscle. The younger animals (4 mo) had lower pH compared to older sheep (8, 12, and 22 mo). In the same study, authors also found that breed affected the ultimate pH of the semitendinosus muscle with the Merino-sired animals having greater pH values compared to Polled Dorset and Border Leicester sheep. In contrast, Teixeira et al. (2005) observed no significant difference in pH of sheep between breeds when measured at 24 h postmortem. Since age and exact breed of DCC and ACC sheep used in the current study was unclear, as no animal trial was performed, we speculated that the pH differences found in this study were most likely due to diet differences. Previous studies have shown that days on pasture has a positive correlation with meat pH in ruminants (McCaughey and Cliplef, 1996; Owens and Gardner, 1999). The branded Dorper sheep meat that the authors purchased for this study was advertised as grass-fed. In addition, sheep production in Australia is primarily based on year-round extensive grazing systems (Ponnampalam et al., 2014), while commercial sheep production in the United States is well known for utilizing a wide range of finishing diets that include cereal grains, oil seeds, and other grain by-products (Stanton and LeValley, 2014).

**Table 1.** Least square means (LSmeans ± standard error of the mean) of objective measurements (Warner Bratzler Shear Force (WBSF), color (CIE L* a* b*)^*a*^ of lean meat, cooking loss and pH) of sheep meat from Dorper, domestic commercial crossbred (DCC), and Australian commercial crossbred (ACC).

	Dorper	DCC	ACC	SEM	*P*-value
Color L*	51.13	51.1	50.74	1.09	0.92
Color a*	12.54	13.77	13.49	1.01	0.45
Color b*	5.49	6.15	6.00	0.46	0.34
Cooking loss, %	15.39^a^	15.17^a^	12.44^b^	1.15	0.02
WBSF, kg	2.16^a^	1.81^b^	1.43^c^	0.10	<0.001
pH	5.94^a^	5.81^b^	5.96^a^	0.04	<0.001

^*a*^ CIE (Commission Internationale de l’Éclairage), L* (lightness), a* (redness), b* (yellowness).

^abc^ LSMeans with a different superscript letter in the same row are significantly different (*P* < 0.05).

In terms of objective color, no differences in CIE L*, a*, or b* values were observed among sheep meat sources (*P* = 0.92, 0.45, 0.34 for L*, a*, b*, respectively; Table 1). These findings contradict other studies that have shown lambs fed a grass/forage diet tended to yield meat that is darker in both lean and fat color than the ones fed on a concentrated diet (Díaz et al., 2002; Priolo et al., 2002, Ekiz et al., 2012). The ultimate pH of meat potentially affects the color of meat (Aberle et al., 2012). Young et al. (1993) reported that sheep meat from Merino breed had a greater pH value (6.37) compared to the sheep meat from other breeds used in said study. However, it is important to note that Young et al. acknowledges that the increase in pH observed in Merino sheep could be attributed to a number of different causes including operator error, variations between sides of the animal and unbalanced groups. While sheep meat from Merino breeds was visually observed during the same study to produce meat of darker color compared to meat from Coopworth breeds, this observation was not quantified instrumentally. In addition to the pH of meat, studies have also shown that degradation of proteins, during the aging process, may affect drip volume and subsequently alter the reflectance of light on meat (Warriss and Brown, 1987). In our study, all the loins were aged extensively for the same period of time (29–32 d). Thus, it is possible that the protein degradation in the aged sheep meat affected the light reflectance, which may explain the lack of difference in L*, a*, and b* values obtained from this study.

The WBSF values were different among the sheep sources *(P <* 0.0001). Dorper breeds had the largest shear force value followed by DCC, with ACC being the most tender out of the three. Belew et al. (2003) determined that WBSF values below 3.2 kg are considered very tender and values above 4.6 kg are considered tough. The WBSF values obtained from this study were all lower than 3.2 kg (Dorper 2.16 kg (1.79–2.89), DCC 1.81 kg (1.14–2.48), and ACC 1.43 kg (1.13–1.78), respectively). These values indicate that sheep meat from all three sources is objectively very tender. There is a multitude of factors such as age, processing conditions, diet, and genetics that can lead to changes in tenderness. On average, U.S. lambs are slaughtered at around 6–8 mo old (USDA FSIS, 2011). On the other hand, lambs are typically slaughtered earlier at around 5 mo of age in Australia (Payne et al., 2020). Although the physiological age of sheep in the current study was unclear, a difference in loin size was observed among sources (Dorper: 3.13 kg, DCC: 3.08 kg, and ACC: 1.95 kg). This difference in age at slaughter between the United States and Australian lambs could be the possible reason for the differences observed in size and tenderness. A study conducted by Bouton et al. (1978) evaluated the shear force values of sheep varying in age from 2–3 mo to 6–8 yr old. They found that as the age of the animal increases, so does the shear force value, indicating that age does in fact impact tenderness. Additionally, the utilization of methods such as electrical stimulation during processing can impact tenderness. Electrical stimulation is often utilized as a means to improve tenderness and prevent meat defects such as cold shortening (Sheridan et al, 1998; Lee et al., 2000). DCC was procured from a large commercial processing plant that regularly utilizes electrical stimulation. On the other hand, the Dorper meat was procured from a small processing plant that likely does not possess this technology. Unfortunately, the authors do not possess knowledge regarding the processing condition of ACC as they were procured from a warehouse. In addition to age and processing conditions, diet can also impact tenderness. Animals fed a grain-based diet have been shown to have improved meat tenderness compared to meat from animals finished on grass (Priolo et al., 2001; Ekiz et al., 2012). As previously stated, the Dorper sheep used in this study were advertised as grass-fed, which could explain the greater WBSF values of Dorper compared to DCC. Although we do not possess the knowledge of the finishing diet for DCC, it is most likely that DCC was supplemented by grains based on the geographical location of lamb source (Northern California) and the month of the year of slaughter (August). Finally, genetics can also play a role in observed changes in tenderness (Fisher et al., 2004; Shackelford et al., 2012). Sañudo et al. (1997) found that the *longissimus lumborum* muscle from Castenella sheep was significantly less tender and less juicy than that from Churra, Manchega, or Awasi crossbreeds at approximately 1 mo of age, suggesting that breed may affect tenderness to some degree. However, the sheep studied by Sañudo may have been too young for meat toughness to develop and thus there may be more pronounced differences with increased age. Factors such as the lack of development of connective tissue and hypertrophy could have masked the tenderness differences among breeds.

Significant differences were found for cooking loss among the different sheep meat sources (*P* = 0.02). Dorper had greater (*P* = 0.01) cooking loss compared to ACC, but was not different from DCC (*P* = 0.85). The cooking loss can be affected by factors like pH, proximate composition, aging time and cooking temperature (Aaslyng et al., 2003; Abdullah and Qudsieh, 2009). Prior studies have shown mixed results on the effect that breed plays in cook loss in other species such as swine (Jeremiah et al., 1999). However, this may not prove true for all species as studies have shown breed to have little to no effect on cooking loss in sheep. Cloete et al. (2012) reported no differences in cooking loss among diverse sheep breeds. Similarly, Van Der Merwe et al. (2020) found that cooking loss did no differ between Dohne Merino, Dormer, Dorper, Meatmaster, Merino, Namaqua Afrikaner, and South African Mutton Merino. Watanabe et al. (2018) concluded that drip loss is negatively correlated with pH, which may explain the differences in cooking loss between Dorper and DCC. However, the lack of difference in cooking loss between Dorper and ACC observed in this study remains unexplained.

### Proximate Composition

The proximate composition of the sheep meat from the three sources is presented in Table 2. No differences in moisture (*P* = 0.64), protein (*P* = 0.10), fat (*P* = 0.46), ash (*P* = 0.31), and calories content (*P* = 0.71)were detected among sheep meat sources. The moisture, fat and crude protein content of sheep meat across all three sources were approximately 71%, 6.1%, and 21% respectively. The low-fat content (5.8–6.6%) observed in each of the three sheep meat sources could be due to the cuts used in this study. Campo et al. (2016) found differing fat content in various cuts of lamb. They found that the breast was the fattest at 42% (only accounting for 4.5% of the overall carcass fat) and the leanest cut was leg at 11.5%.

**Table 2.** Least squares means (LSMeans) of proximate composition (g/100 g) of sheep meat from Dorper, domestic commercial crossbred (DCC), and Australian commercial crossbred (ACC)

	Dorper	DCC	ACC	SEM	*P*-value
Moisture	70.94	70.83	70.48	0.50	0.65
Protein	21.35	21.20	21.72	0.25	0.10
Fat	5.83	6.65	5.86	0.74	0.46
Ash	0.91	0.90	0.85	0.05	0.31
Carbohydrates	1.09^a^	0.55^b^	1.13^a^	0.26	0.05
Calories, cal	142.1	146.85	144.2	5.73	0.71

^ab^ LSMeans with a different superscript letter in the same row are significantly different (*P* < 0.05).

The Dorper samples had greater carbohydrate content compared to DCC (*P* = 0.04), but did not differ from ACC (*P* = 0.86) based on carbohydrate by difference. The concentration of glycogen, the primary form of carbohydrates in meat/muscles may be altered by factors such as diet, lairage time, transportation stress, and processing technology. These factors may affect the glycogen content, a major storage compound of carbohydrates in the muscle (Rosenvold et al., 2002; Ferguson and Warner, 2008; Díaz et al., 2014) through direct means, or indirectly by influencing glucose concentration (Liste et al., 2011; Bernardini et al., 2012). For example, Santé -Lhoutellier et al. (2008) investigated the effect of grass-fed and concentrate-fed diets on glycogen content of the *longissimus dorsi* muscle from sheep and found that animals fed with concentrate diets had greater glycogen levels compared to grass-fed animals. Similar to the current study, no color difference in sheep meet was observed between the grass and concentrate fed lambs. Although no difference in color was observed in the current study, it is important to note that a similar trend was seen in carbohydrate content and pH. As previously mentioned, there is a likelihood that ACC and Dorper sheep used in this study were raised on a grass-only diet while the DCC sheep were supplemented by grain. However, changes in glycogen levels resulting in color change cannot be attributed to diet alone but rather to a combination of additional factors (Ponnampalam et al., 2017).

### Consumer Demographics

The demographic data of 120 consumers that participated in the sensory panel evaluation of the three sheep sources are presented in Table 3. More female (55.8%) than male (43.4%) consumers participated in the consumer sensory panel. The majority of participants were either Asian (48.3%) or Caucasian (32.5%). Most participants were recruited from the University of California-Davis campus, and thus a large number of the consumers were between 20 and 29 yr old (70.8%), and 100% of the consumers had at least some college/technical school experience. This also helps explain why only 31.8% of the consumers had a yearly household income above US$75,000. More than 40% of the consumers reported eating meat multiples times per day (43.7%) and consuming sheep at least once a month (45.8%). Lastly, quality (54.9%) and price (38.2%) were the most important factors that influenced the consumers’ decision to purchase sheep meat.

**Table 3.** Demographic data of consumers (*n* = 120) that participated in sensory panel evaluation of sheep meat from Dorper, domestic commercial crossbred (DCC), and Australian commercial crossbred (ACC).

Characteristic	Response	Percentage of responders (%)
Gender	Male	43.3
	Female	55.8
	Other	0.8
Ethnic origin	Caucasian	32.5
	African American	0
	African American/Asian	0.8
	Hispanic	5
	Asian	48.3
	Middle Eastern	4.1
Age	Under 20	10
	20–29	70.8
	30–39	11.6
	40–49	0.8
	50–59	1.6
	Over 60	5
Education Level	Some college/technical school	29.1
	College graduate	31.6
	Post graduate	34.1
	Others	0
Household size	1 People	21.6
	2 People	16.6
	3 People	25
	4 People	24.1
	5 People	6.6
	6 People	3.3
	Over 6 People	2.5
Yearly household income	Less than $20,000 $20,000 to $34,999 $35,000 to $49,999 $50,000 to $74,999 $75,000 to $99,999 $100,000 or more	22.6 29.4 10.1 5.8 9.2 22.6
Frequency of meat consumption	Multiple times a day Once a day Several times a week Once a week Less than once a week	43.6 21.8 26.8 6.7 0.8
Frequency of lamb meat consumption	At least once a week At least once a month At least once every 6 mo At least once a year	8.3 45.8 29.1 16.6
Important factor that influence lamb meat purchase	Quality Price Country of origin Part of the lamb Don’t buy lamb	54.9 38.2 2.9 2.9 0.9

### Consumer Panel

The consumer panel results are presented in Tables 4a–4d. Dorper was rated to have a lower tenderness liking score than ACC *(P* = 0.0087), but was not rated differently from DCC by consumers (*P* = 0.76). This result was supported by the WBSF values obtained in the current study (Table 1), where ACC had the lowest WBSF values among the three sources. Discussion regarding potential differences in tenderness among different treatments in this study was also provided in the same section.

**Table 4a.** Consumer tasting panel data for tenderness of Dorper, domestic commercial crossbred (DCC), and Australian commercial crossbred (ACC) sheep meat^*a*^

	Dorper	DCC	ACC
1 – Dislike extremely	0.62%	0.62%	0.62%
2	3.75%	2.5%	1.88%
3	8.12%	7.5%	5.63%
4	7.5%	8.13%	6.25%
5	5%	3.75%	3.12%
6	21.25%	16.25%	14.38%
7	26.88%	23.75%	28.12%
8	23.75%	30%	30%
9 – Like extremely	3.13%	7.5%	10%
Means with SE	6.21(0.14)^b^	6.50 (0.15)^ab^	6.75 (0.14)^a^
*P*-value^*b*^	0.011		

^*a*^ A 9-point hedonic scale was used for the consumer tasting panels (1 = dislike extremely, 2 = dislike very much, 3 = dislike moderately, 4 = dislike slightly, 5 = neither like nor dislike, 6 = like slightly, 7 = like moderately, 8 = like very much, and 9 = like extremely).

^*b*^ Kruskal–Wallis and Dwass–Steel–Critchlow–Fligner (post hoc pairwise multiple comparison) were used to determine the significance of the sheep sources. *P*-value is from Kruskal–Wallis test.

^abc^ Significantly different from one sheep source to another sheep source (*P* < 0.05).

**Table 4b.** Consumer tasting panel data for juiciness of Dorper, domestic commercial crossbred (DCC), and Australian commercial crossbred (ACC) sheep meat^*a*^

	Dorper	DCC	ACC
1 – Dislike extremely	1.25%	1.87%	1.25%
2	3.12%	1.87%	4.38%
3	7.5%	5.63%	2.5%
4	10.63%	10.63%	10.63%
5	13.13%	10%	7.5%
6	24.38%	21.87%	20.62%
7	16.87%	16.88%	27.5%
8	18.75%	25.63%	20%
9 – Like extremely	4.37%	5.62%	5.62%
Means with SE	5.91 (0.15)	6.20 (0.15)	6.24 (0.14)
*P*-value^*b*^	0.151		

^*a*^ A 9-point hedonic scale was used for the consumer tasting panels (1 = dislike extremely, 2 = dislike very much, 3 = dislike moderately, 4 = dislike slightly, 5 = neither like nor dislike, 6 = like slightly, 7 = like moderately, 8 = like very much, and 9 = like extremely).

^*b*^ Kruskal–Wallis and Dwass–Steel–Critchlow–Fligner (post hoc pairwise multiple comparison) were used to determine the significance of the sheep sources. *P*-value is from Kruskal–Wallis test.

^abc^ Significantly different from one sheep source to another sheep source (*P* < 0.05).

**Table 4c.** Consumer tasting panel data for flavor of Dorper, domestic commercial crossbred (DCC), and Australian commercial crossbred (ACC) sheep meat^*a*^

	Dorper	DCC	ACC
1 – Dislike extremely	2.5%	0.62%	0%
2	8.12%	1.88%	5%
3	11.87%	6.25%	5.63%
4	11.87%	12.5%	13.13%
5	8.13%	15.63%	11.25%
6	18.13%	13.75%	24.38%
7	21.88%	21.88%	23.75%
8	13.13%	21.87%	14.38%
9 – Like extremely	4.37%	5.62%	1.88%
Means with SE	5.48 (0.17)^b^	6.12 (0.14)^a^	5.83 (0.14)^ab^
*P*-value^*b*^	0.026		

^*a*^ A 9-point hedonic scale was used for the consumer tasting panels (1 = dislike extremely, 2 = dislike very much, 3 = dislike moderately, 4 = dislike slightly, 5 = neither like nor dislike, 6 = like slightly, 7 = like moderately, 8 = like very much, and 9 = like extremely).

^*b*^ Kruskal–Wallis and Dwass–Steel–Critchlow–Fligner (post hoc pairwise multiple comparison) were used to determine the significance of the sheep sources. *P*-value is from Kruskal–Wallis test.

^abc^ Significantly different from one sheep source to another sheep source (*P* < 0.05).

**Table 4d.** Consumer tasting panel data for overall acceptance of Dorper, domestic commercial crossbred (DCC), and Australian commercial crossbred (ACC) sheep meat^*a*^

	Dorper	DCC	ACC
1 – Dislike extremely	2.5%	0.625%	0%
2	5.63%	1.25%	4.37%
3	10%	5%	4.37%
4	13.13%	14.375%	15%
5	10%	8.75%	6.25%
6	20.62%	17.5%	19.38%
7	18.12%	25.625%	31.87%
8	16.87%	20.625%	13.75%
9 – Like extremely	3.12%	6.25%	5%
Means with SE	5.6 (0.16)^b^	6.25 (0.14)^a^	6.07 (0.14)^a^
*P*-value^*b*^	0.013		

^*a*^ A 9-point hedonic scale was used for the consumer tasting panels (1 = dislike extremely, 2 = dislike very much, 3 = dislike moderately, 4 = dislike slightly, 5 = neither like nor dislike, 6 = like slightly, 7 = like moderately, 8 = like very much, and 9 = like extremely).

^*b*^ Kruskal–Wallis and Dwass–Steel–Critchlow–Fligner (post hoc pairwise multiple comparison) were used to determine the significance of the sheep sources. *P*-value is from Kruskal–Wallis test.

^abc^ Significantly different from one sheep source to another sheep source (*P* < 0.05).

Dorper was rated with lower flavor acceptability compared to DCC sheep meat (*P* = 0.02), but was not different from ACC in the consumer panel (*P* = 0.32). The flavor development of meat is the direct result of interactions between lipids, amino acids, sugar, and heat to generate volatile compounds. This process can be influenced by different factors such as breed, sex, and animal diet, which may affect the proximate composition and pH of meat (Field, 1984; Mottram and Salter, 1989; Elmore et al., 2000). In this study, we noticed similar patterns in pH, carbohydrate content and flavor acceptability across the three sheep sources. Young et al. (1993) showed that greater pH value adversely affected sheep meat flavor. The lower flavor acceptability of Dorper meat may be due to diet. These findings are in agreement with a study conducted by O’Reilly et al. (2020) who studied the impact of various demographic factors of American, Australian, and Chinese consumers on sensory scoring of sheep meat. They found that two of the most impactful demographic factors were that of age and income. In Chinese consumers, they found that older consumers were more generous in their scoring of lamb. In this study, the vast majority of the consumers in the panel identified as being between 20 and 29 yr old (70.8%).

No difference (*P* = 0.15) in juiciness among the three sheep meat sources was detected by consumers. However, Hoffman et al. (2003), reported juiciness differences between two sheep breeds of the six breeds tested, suggesting that breed may affect juiciness at least to some degree. In addition, Priolo et al. (2002) reported sheep meat from grain-fed sheep was juicier than sheep meat from grass-fed sheep. Finally, juiciness has been shown to correlate with cooking loss (Safari et al., 2001). Our results somewhat contradicted the results from other studies as we reported differences in cooking loss among the three sheep sources, but no difference in juiciness was detected by the consumer panel. One possible explanation for this discrepancy is that there was no difference in fat content among groups. The juiciness of meat is highly impacted by fat content (O’Quinn et al., 2012; Listrat et al., 2016). Since there was no difference in fat content among groups it is expected that there would also be no difference in juiciness as well. However, it is important to note that most of the differences in juiciness observed in prior studies were identified by trained panelists who might be able to identify differences in juiciness that consumers were not able to identify.

Dorper was rated with lower overall acceptance scores by the consumers compared to DCC (*P* = 0.01) and rated similar to ACC (*P* = 0.14). Safari et al. (2001) reported that tenderness and flavor are the two most important sensory attributes of meat that influence the overall acceptability of meat by consumers. All things considered, Dorper was rated with the lowest overall acceptance score by the consumers this could be attributed to lower tenderness and a lower level of flavor acceptability. This result was supported by Navajas et al. (2008), who reported that Scottish Blackface sheep meat had greater overall liking compared to Texel sheep due to greater tenderness and flavor ratings. However, consumer demographics may also influence consumer acceptance. O’Reilly et al. (2020) found that income played a significant role in consumers’ rating of sheep meat. They found that those in the lower-income brackets tended to rate the tenderness, flavor, and overall likeness of lamb higher than those in higher income brackets. In this study over half (67.9%) of consumers reported a yearly household income below $75,000 which could influence their overall acceptance.

### Trained Sensory Panel

The data of the trained panel are presented in Table 5. The trained panelists rated Dorper meat less tender than ACC (*P* = 0.002), but not different from DCC (*P* = 0.10). These results further corroborate our data from the consumer panels and were supported by the WBSF values, which all showed Dorper to be the least tender sheep source out of the three.

**Table 5.** Least squares means (LSMeans) of three sensory attributes (intensity of flavor, tenderness and off-flavor) of Dorper, domestic commercial crossbred (DCC), and Australian commercial crossbred (ACC) sheep meat assessed in the trained panel evaluation^*a*^

	Dorper	DCC	ACC	SEM	*P*-value
Flavor	32.14	29.03	28.93	2.23	0.28
Tenderness	76.31^b^	79.27^ab^	81.83^a^	1.72	0.01
Off-Flavor	14.47^a^	10.39^b^	13.56^a^	1.21	0.02

^*a*^ An unstructured line scale anchored at both ends was used (0 = absence or low intensity, 100 = extreme intensity).

^abc^ LSMeans with a different superscript letter in the same row are significantly different (*P* < 0.05).

No differences (*P* = 0.46) in lamb flavor intensity among the three sheep sources were identified by the trained panelists. However, trained panelists reported Dorper (*P* = 0.009) and ACC (*P* = 0.03) had greater off-flavor intensity compared to DCC. Soapy, earthy and serum off-flavors were specifically noted by trained panelists for Dorper, and mutton and oxidized off-flavors were reported for ACC. Additionally, trained panelists identified grassy, liver, metallic, fishy and sour off-flavors for all the sheep meat sources. Kemp et al., (1980) and Rousset-Akrim et al., (1997) demonstrated that nutritional regimen before harvest had a strong impact on flavor and off-flavor ratings of sheep meat. Sañudo et al. (2000) further showed that finishing diet plays a more important role than breeds in determining the final flavor and off-flavor intensity of sheep meat as they demonstrated that the mutton off-flavor intensity was similar across Romney, Hampshire, Columbia, Rambooillet and Merino breeds when they were on a similar diet. Finally, Duckett and Kuber (2001) concluded that finishing sheep on pasture increased the intensity of off-flavors. As discussed previously, it is highly likely that the finishing diet for ACC sheep and Dorper sheep was grass while DCC sheep were finished on grain, which explains the greater intensity of off-flavor in ACC and Dorper meat evaluated by trained panelists.

## CONCLUSION

The study compared the quality, proximate composition and sensory attributes among Dorper, DCC and ACC. The results indicated that there were apparent meat quality differences among the three sheep meat sources. Differed from common perception, DCC was preferred over Dorper and ACC by consumers and trained panelists. Additional research with a more controlled environment is needed to shed light on the quality and palatability traits of Dorper.

## References

[CIT0001] Aaslyng, M. D., C. Bejerholm, P. Ertbjerg, H. C. Bertram, and H. J. Andersen. 2003. Cooking loss and juiciness of pork in relation to raw meat quality and cooking procedure. Food Qual. Prefer. 14:277–288. doi:10.1016/S0950-3293(02)00086-1

[CIT0002] Abdullah, A. Y., and R. I. Qudsieh. 2009. Effect of slaughter weight and aging time on the quality of meat from Awassi ram lambs. Meat Sci. 82:309–316. doi:10.1016/j.meatsci.2009.01.027 20416727

[CIT0003] Aberle, E. D., J. C. Forrest, D. E. Gerrard, and E. W. Mills. 2012. Conversion of muscle to meat: biochemistry of meat quality development. In: Principles of meat science. 5th ed. Dubuque, IA: Kendall Hunt Publishing; p. 127.

[CIT0004] American Meat Science Association. 2019. Research guidelines for cookery, sensory evaluation, and instrumental tenderness measurements of meat. Available from https://meatscience.org/publications-resources/printed-publications/sensory-and-tenderness-evaluation-guidelines [accessed February 16, 2020].

[CIT0005] AOAC International. 1997. Official methods of analysis of AOAC International. 16th ed. Vol. 1. Arlington, VA: AOAC International.

[CIT0006] Basson, W. D., B. D. Van Niekerk, A. M. Mulder, and J. G. Cloete. 1969. The productive and reproductive potential of three breeds mated at 8-monthly intervals under intensive feeding conditions. In: H.S. Hofmeyer, editor. Proceedings of the South African Society for Animal Production. South Africa: South African Society for Animal Science Inc.; p. 149–154.

[CIT0007] Belew, J. B., J. C. Brooks, D. R. McKenna, and J. W. Savell. 2003. Warner-Bratzler shear evaluations of 40 bovine muscles. Meat Sci. 64:507–512. doi:10.1016/S0309-1740(02)00242-5 22063134

[CIT0008] Bernardini, D., G. Gerardi, A. Peli, L. Nanni Costa, M. Amadori, and S. Segato. 2012. The effects of different environmental conditions on thermoregulation and clinical and hematological variables in long-distance road-transported calves. J. Anim. Sci. 90:1183–1191. doi:10.2527/jas.2011-4113 22100587

[CIT0009] Bouton, P. E., P. V. Harris, D. Ratcliff, and D. W. Roberts. 1978. Shear force measurements on cooked meat from sheep of various ages. J. Food Sci. 43:1038–1039. doi:10.1111/j.1365-2621.1978.tb02486.x

[CIT0010] Campo, M. M., E. Muela, V. C. Resconi, M. Barahona, and C. Sañudo. 2016. Influence of commercial cut on proximate composition and fatty acid profile of Rasa Aragonesa light lamb. J. Food Compos. Anal. 53:7–12. doi:10.1016/j.jfca.2016.08.001

[CIT0011] Canton, G. J., Q. R. Bores, R. J. Baeza, F. J. Quintal, R. R. Santos, and C. C. Sandoval. 2009. Growth and feed efficiency of pure and F1 pelibuey lambs crossbred with specialized breeds for production of meat. J. Anim. Vet. Adv. 8:26–32.

[CIT0012] Cheng, P., 1984. Livestock breeds of China. Food and Agriculture Organization of the United Nations. FAO Animal Production and Health Paper, 46. Beijing, China: China Academic Publishers.

[CIT0013] Clarke, J. N., C. A. Morris, P. A. Speck, G. C. Upreti, and G. B. Nicoll. 1996. Genetic improvement of meat quality in sheep and cattle. In: S. Peterson, editor. Proceedings New Zealand Society of Animal Production. New Zealand: New Zealand Society of Animal Production; p. 157–161.

[CIT0014] Cloete, J. J., L. C. Hoffman, and S. W. Cloete. 2012. A comparison between slaughter traits and meat quality of various sheep breeds: wool, dual-purpose and mutton. Meat Sci. 91:318–324. doi:10.1016/j.meatsci.2012.02.010 22391055

[CIT0015] Deng, K. D., Q. Y. Diao, C. G. Jiang, Y. Tu, N. F. Zhang, J. Liu, T. Ma, Y. G. Zhao, and G. S. Xu. 2012. Energy requirements for maintenance and growth of Dorper crossbred ram lambs. Livest. Sci. 150:102–110. doi:10.1016/j.livsci.2012.08.006

[CIT0016] Díaz, M. T., S. Velasco, V. Cañeque, S. Lauzurica, F. Ruiz De Huidobro, C. Pérez, J. González, and C. Manzanares. 2002. Use of concentrate or pasture for fattening lambs and its effect on carcass and meat quality. Small Rumin Res. 43:257–268. doi:10.1016/S0921-4488(02)00016-0

[CIT0017] Díaz, M. T., C. Vieira, C. Pérez, S. Lauzurica, E. G. de Chávarri, M. Sánchez, and J. De la Fuente. 2014. Effect of lairage time (0h, 3 h, 6 h or 12 h) on glycogen content and meat quality parameters in suckling lambs. Meat Sci. 96(2 Pt A):653–660. doi:10.1016/j.meatsci.2013.10.013 24200553

[CIT0018] Duckett, S. K., and P. S. Kuber. 2001. Genetic and nutritional effects on lamb flavor. J. Anim. Sci. 79:249–254. doi:10.2527/jas2001.79e-supple249x

[CIT0019] Ekiz, B., A. Yilmaz, M. Ozcan, and O. Kocak. 2012. Effect of production system on carcass measurements and meat quality of Kivircik lambs. Meat Sci. 90:465–471. doi:10.1016/j.meatsci.2011.09.008 21978412

[CIT0020] Elmore, J. S., D. S. Mottram, M. Enser, and J. D. Wood. 2000. The effects of diet and breed on the volatile compounds of cooked lamb. Meat Sci. 55:149–159. doi:10.1016/s0309-1740(99)00137-0 22061080

[CIT0021] England, E., 2018. 11 Factors affecting the transformation of muscle to meat. J. Anim. Sci. 96:495. doi:10.1093/jas/sky404.1081

[CIT0022] Ferguson, D. M., and R. D. Warner. 2008. Have we underestimated the impact of pre-slaughter stress on meat quality in ruminants? Meat Sci. 80:12–19. doi:10.1016/j.meatsci.2008.05.004 22063165

[CIT0023] Field, R. A. 1984. Consumer acceptance of ram and wether meat in the USA. In: Proceedings of New Zealand Society of Animal Production. New Zealand Society of Animal Production, New Zealand; p. 209–210.

[CIT0024] Fisher, M., S. Duckett, and M. Wildeus. 2004. Effect of breed type, finishing treatment, and dietary supplements on carcass characteristics and tenderness in hair sheep. J. Anim. Sci. 82:227–228.

[CIT0025] Font-i-Furnols, M., and L. Guerrero. 2014. Consumer preference, behavior and perception about meat and meat products: an overview. Meat Sci. 98:361–371. doi:10.1016/j.meatsci.2014.06.025 25017317

[CIT0026] Hoffman, L. C., M. Muller, S. W. Cloete, and D. Schmidt. 2003. Comparison of six crossbred lamb types: sensory, physical and nutritional meat quality characteristics. Meat Sci. 65:1265–1274. doi:10.1016/S0309-1740(03)00034-2 22063769

[CIT0027] Hopkins, D. L., D. F. Stanley, L. C. Martin, E. S. Toohey, and A. R. Gilmour. 2007. Genotype and age effects on sheep meat production. 3. Meat quality. Aust. J. Exp. Agric. 47:1155–1164. doi:10.1071/EA06299

[CIT0028] Jeremiah, L. E., J. P. Gibson, L. L. Gibson, R. O. Ball, C. Aker, and A. Fortin. 1999. The influence of breed, gender, and PSS (halothane) genotype on meat quality, cooking loss, and palatability of pork. Food Res. Int. 32:59–71. doi:10.1016/S0963-9969(99)00077-0

[CIT0029] Jones, K. G. 2004. Trends in the US sheep industry. USDA Economic Research Service, Agricultural Information Bulletin 33681. Available from https://downloads.usda.library.cornell.edu/usda-esmis/files/kw52j804p/08612r694/gm80hz92n/aib787.pdf. [accessed February 22, 2021].

[CIT0030] Kemp, J. D., M. Mahyuddin, D. G. Ely, J. D. Fox, and W. G. Moody. 1980. Effect of feeding systems, slaughter weight and sex on organoleptic properties, and fatty acid composition of lamb. J. Anim. Sci. 51:321–330. doi:10.2527/jas1980.512321x

[CIT0031] Lee, S., P. Polidori, R. G. Kauffman, and B. C. Kim. 2000. Low-voltage electrical stimulation effects on proteolysis and lamb tenderness. J Food Sci. 65:786–790. doi:10.1111/j.1365-2621.2000.tb13587.x

[CIT0032] Liste, G., G. C. Miranda-De La Lama, M. M. Campo, M. Villarroel, E. Muela, and G. A. Mara. 2011. Effect of lairage on lamb welfare and meat quality. Anim. Prod. Sci. 51:952–958. doi:10.1071/AN10274

[CIT0033] Listrat, A., B. Lebret, I. Louveau, T. Astruc, M. Bonnet, L. Lefaucheur, B. Picard, and J. Bugeon. 2016. How muscle structure and composition influence meat and flesh quality. Scientific World J. 2016:3182746. doi:10.1155/2016/3182746 PMC4789028 27022618

[CIT0034] Ma, T., K. D. Deng, Y. Tu, C. G. Jiang, N. F. Zhang, Y. L. Li, B. W. Si, C. Lou, and Q.Y. Diao. 2014. Effect of dietary concentrate:forage ratios and undegraded dietary protein on nitrogen balance and urinary excretion of purine derivatives in Dorper × thin-tailed Han crossbred lambs. Asian-Australas. J. Anim. Sci. 27:161–168. doi:10.5713/ajas.2013.13338 25049939 PMC4093213

[CIT0035] Manyuchi, B., H. P. Tawonezvi, and R. M. Chiwara. 1991. Breed and environmental influences on weaner lamb production in Zimbabwe. Trop. Anim. Health Prod. 23:115–125. doi:10.1007/BF02361196 1858164

[CIT0036] McCaughey, W. P., and R. L. Cliplef. 1996. Carcass and organoleptic characteristics of meat from steers grazed on alfalfa/grass pastures and finished on grain. Can. J. Anim. Sci. 76:149–152.

[CIT0037] Merrill, A. L., and B. K. Watt. 1973. Energy value of foods: basis and derivation, revised. U.S. Department of Agriculture, Agriculture Handbook 74. Available from https://www.ars.usda.gov/ARSUserFiles/80400525/Data/Classics/ah74.pdf. [accessed February 22, 2021].

[CIT0038] Monro, J., and B. Burlingame. 1996. Carbohydrates and related food components: INFOODS tagnames, meanings, and uses. J Food Compos Anal. 9:100–118. doi:10.1006/jfca.1996.0018

[CIT0039] Mottram, D. S., and L. J. Salter. 1989. Flavor formation in meat-related Maillard systems containing phospholipids. In: Thermal generation of aromas. Washington DC: American Chemical Society; p. 442–451.

[CIT0040] Navajas, E. A., N. R. Lambe, A. V. Fisher, G. R. Nute, L. Bünger, and G. Simm. 2008. Muscularity and eating quality of lambs: effects of breed, sex and selection of sires using muscularity measurements by computed tomography. Meat Sci. 79:105–112. doi:10.1016/j.meatsci.2007.08.006 22062603

[CIT0041] O’Quinn, TG., Brooks JC, Polkinghorne RJ, Garmyn AJ, Johnson BJ, Starkey JD, Rathmann RJ, Miller MF. 2012. Consumer assessment of beef strip loin steaks of varying fat levels. J Anim Sci. 90:626–634. doi:10.2527/jas.2011-4282 21948609

[CIT0042] O’Reilly, R. A., L. Pannier, G. E. Gardner, A. J. Garmyn, H. Luo, Q. Meng, M. F. Miller, and D. W. Pethick. 2020. Influence of demographic factors on sheepmeat sensory scores of American, Australian and Chinese consumers. Foods. 9:529. doi:10.3390/foods9040529 PMC7230945 32331353

[CIT0043] Owens, F. N., and B. A. Gardner. 1999. Ruminant nutrition and meat quality. In: 52nd Reciprocal Meat Conference. Chicago, IL, p. 25–36.

[CIT0044] Payne, C. E., L. Pannier, F. Anderson, D. W. Pethick, and G. E. Gardner. 2020. Lamb age has little impact on eating quality. Foods 9:187. doi:10.3390/foods9020187 PMC7073923 32069988

[CIT0045] Ponnampalam, E. N., K. L. Butler, R. H. Jacob, D. W. Pethick, A. J. Ball, J. E. Edwards, G. Geesink, and D. L. Hopkins. 2014. Health beneficial long chain omega-3 fatty acid levels in Australian lamb managed under extensive finishing systems. Meat Sci. 96(2 Pt B):1104–1110. doi:10.1016/j.meatsci.2013.04.007 23643471

[CIT0046] Ponnampalam, E. N., D. L. Hopkins, H. Bruce, D. Li, G. Baldi, and A. E. Bekhit. 2017. Causes and contributing factors to “Dark Cutting” meat: current trends and future directions: a review. Compr. Rev. Food Sci. Food Saf. 16:400–430. doi:10.1111/1541-4337.12258 33371557

[CIT0047] Priolo, A., D. Micol, and J. Agabriel. 2001. Effects of grass feeding systems on ruminant meat colour and flavour. A review. Anim Res. 50:185–200. doi:10.1051/animres:2001125

[CIT0048] Priolo, A., D. Micol, J. Agabriel, S. Prache, and E. Dransfield. 2002. Effect of grass or concentrate feeding systems on lamb carcass and meat quality. Meat Sci. 62:179–185. doi:10.1016/s0309-1740(01)00244-3 22061409

[CIT0049] Rosenvold, K., H. N. Lærke, S. K. Jensen, A. H. Karlsson, K. Lundström, and H. J. Andersen. 2002. Manipulation of critical quality indicators and attributes in pork through vitamin E supplementation, muscle glycogen reducing finishing feeding and pre-slaughter stress. Meat Sci. 62:485–496. doi:10.1016/s0309-1740(02)00045-1 22061757

[CIT0050] Rousset-Akrim, S., O. A. Young, and J. L. Berdagué. 1997. Diet and growth effects in panel assessment of sheepmeat odour and flavour. Meat Sci. 45:169–181. doi:10.1016/s0309-1740(96)00099-x 22061301

[CIT0051] Russell, B. C., G. McAlister, I. S. Ross, and D. W. Pethick. 2005. Lamb and sheep meat eating quality – industry and scientific issues and the need for integrated research. Aust. J. Exp. Agric. 45:465–467. doi:10.1071/EA04007

[CIT0052] Safari, E., N. M. Fogarty, G. R. Ferrier, L. D. Hopkins, and A. Gilmour. 2001. Diverse lamb genotypes. 3. Eating quality and the relationship between its objective measurement and sensory assessment. Meat Sci. 57:153–159. doi:10.1016/s0309-1740(00)00087-5 22061358

[CIT0053] Santé-Lhoutellier, V., E. Engel, and P. Gatellier. 2008. Assessment of the influence of diet on lamb meat oxidation. Food Chem. 109:573–579. doi:10.1016/j.foodchem.2007.11.081

[CIT0054] Sañudo, C., M. M. Campo, I. Sierra, G. A. María, J. L. Olleta, and P. Santolaria. 1997. Breed effect on carcase and meat quality of suckling lambs. Meat Sci. 46:357–365. doi:10.1016/s0309-1740(97)00030-2 22062319

[CIT0055] Sañudo, C., M. E. Enser, M. M. Campo, G. R. Nute, G. María, I. Sierra, and J. D. Wood. 2000. Fatty acid composition and sensory characteristics of lamb carcasses from Britain and Spain. Meat Sci. 54:339–346. doi:10.1016/s0309-1740(99)00108-4 22060790

[CIT0056] Shackelford, S. D., K. A. Leymaster, T. L. Wheeler, and M. Koohmaraie. 2012. Effects of breed of sire on carcass composition and sensory traits of lamb. J. Anim. Sci. 90:4131–4139. doi:10.2527/jas.2012-5219 22665668

[CIT0057] Sheridan, J. J., B. McGeehin, and F. Butler. 1998. Effects of ultra-rapid chilling and electrical stimulation on the tenderness of lamb carcass muscles. J. Muscle Foods. 9:403–417. doi:10.1111/j.1745-4573.1998.tb00744.x

[CIT0058] Snowder, G. D., and S. K. Duckett. 2003. Evaluation of the South African Dorper as a terminal sire breed for growth, carcass, and palatability characteristics. J. Anim. Sci. 81:368–375. doi:10.2527/2003.812368x 12643479

[CIT0059] Souza, D. A., A. B. Selaive-Villarroel, E. S. Pereira, E. M. C. Silva, and R. L. Oliveira. 2016. Effect of the Dorper breed on the performance, carcass and meat traits of lambs bred from Santa Inês sheep. Small Rumin. Res. 145:76–80. doi:10.1016/j.smallrumres.2016.10.017

[CIT0060] Stanton, T. L., and S. B. LeValley. 2014. Lamb Feedlot Nutrition. Colorado State University Extension, Fact Sheet No. 1.613. Available from https://extension.colostate.edu/topic-areas/agriculture/lamb-feedlot-nutrition-1-613/ [accessed February 22, 2021].

[CIT0061] Teixeira, A., S. Batista, R. Delfa, and V. Cadavez. 2005. Lamb meat quality of two breeds with protected origin designation. Influence of breed, sex and live weight. Meat Sci. 71:530–536. doi:10.1016/j.meatsci.2005.04.036 22060929

[CIT0062] USDA. 2015. National nutrient database for standard reference, release 28 (slightly revised). Version Current: May 2016. Internet: http://www.ars.usda.gov/ba/bhnrc/ndl

[CIT0063] USDA Agricultural Research Service. 2015. Slice shear force protocol for AMSA sensory guidelines. Available from https://meatscience.org/docs/default-source/publications-resources/amsa-sensory-and-tenderness-evaluation-guidelines/research-guide-support-documents/ssf-protocol-for-amsa-sensory-guidelines.pdf?sfvrsn=bbe984b3_8 [accessed February 16, 2020].

[CIT0064] USDA Economic Research Service. 2016. Retail prices for beef, pork, poultry cuts, eggs, and dairy products. USDA Economic Research Service. Available from https://www.ers.usda.gov/data-products/meat-price-spreads/ [accessed February 22, 2021].

[CIT0065] USDA Economic Research Service. 2019. Sector at a glance—U. S. Production. Available from https://www.ers.usda.gov/topics/animal-products/sheep-lamb-mutton/sector-at-a-glance/ [accessed February 16, 2020].

[CIT0066] USDA Food Safety and Inspection Service. 2011. Lamb from farm to table. Available from https://permanent.fdlp.gov/gpo19083/Lamb-from-Farm-to-Table.pdf [accessed October 30, 2020].

[CIT0067] Van Der Merwe, D. A., T. S. Brand, and L. C. Hoffman. 2020. Slaughter characteristics of feedlot-finished premium South African lamb: effects of sex and breed type. Foods. 9:648. doi:10.3390/foods9050648 PMC7278794 32443429

[CIT0068] Warriss, P.D., and S.N. Brown. 1987. The relationships between initial pH, reflectance and exudation in pig muscle. Meat Sci. 20:65–74. doi:10.1016/0309-1740(87)90051-9 22056120

[CIT0069] Watanabe, G., M. Motoyama, I. Nakajima, and K. Sasaki. 2018. Relationship between water-holding capacity and intramuscular fat content in Japanese commercial pork loin. Asian-Australas. J. Anim. Sci. 31:914–918. doi:10.5713/ajas.17.0640 29268572 PMC5933991

[CIT0070] Yeaman, J. C., D. F. Waldron, and T. D. Willingham. 2013. Growth and feed conversion efficiency of Dorper and Rambouillet lambs. J. Anim. Sci. 91:4628–4632. doi:10.2527/jas.2012-6226 23893989

[CIT0071] Young, O. A., D. H. Reid, and G. H. Scales. 1993. Effect of breed and ultimate pH on the odour and flavour of sheep meat. New Zeal. J. Agric. Res. 3:363–370. doi:10.1080/00288233.1993.10417733

